# *Rickettsia lanei* Rickettsiosis, Oregon, USA, 2025

**DOI:** 10.3201/eid3204.251962

**Published:** 2026-04

**Authors:** Stephen G. Ladd-Wilson, Richard W. Fawcett, Sarah Y. Park, Shivkumar Venkatasubrahmanyam, Martin S. Lindner, Scott Davis, Alanna Spry, Joseph Singleton, Sandor E. Karpathy, Christopher D. Paddock

**Affiliations:** Public Health Division, Oregon Health Authority, Portland, Oregon, USA (S.G. Ladd-Wilson); Columbia Sands Infectious Diseases, Redmond, Oregon, USA (R.W. Fawcett); Karius., Inc., Redwood City, California, USA (S.Y. Park, S. Venkatasubrahmanyam, M.S. Lindner); Home Town Animal Hospital, Prineville, Oregon, USA (S. Davis); Crook County Health Department, Prineville (A. Spry); Centers for Disease Control and Prevention, Atlanta, Georgia, USA (J. Singleton, S.E. Karpathy, C.D. Paddock)

**Keywords:** rickettsiosis, Rickettsia lanei, bacteria, vector-borne diseases, Oregon, United States

## Abstract

Using metagenomic sequencing, we identified a patient infected with *Rickettsia lanei* who was initially diagnosed with Rocky Mountain spotted fever (RMSF), a clinically similar disease caused by infection with *R. rickettsii*. Our investigation highlights the importance of clinical, epidemiologic, and laboratory partnerships to leverage the discovery of novel pathogens.

Spotted fever group rickettsioses (SFGRs) comprise multiple febrile, rash-associated illnesses caused by various arthropodborne, intracellular *Rickettsia* bacteria. Rocky Mountain spotted fever (RMSF), the most severe SFGR, was first described in the western United States around the turn of the 20th Century (https://www.niaid.nih.gov/diseases-conditions/rocky-mountain-spotted-fever). For the next ≈100 years, RMSF was considered the only tickborne SFGR in the United States; however, since 2004, investigators have identified additional pathogens causing SFGRs of varying severity that share clinical features with RMSF (*1*–*3*). Cases of RMSF have been described in Oregon, USA, since 1903 (*4*,*5*). We describe an Oregon patient infected with the recently characterized pathogen *Rickettsia lanei* whose illness resembled RMSF.

In July 2025, a >50-year-old man sought care at an emergency department for 7 days of fever, headache, and myalgias that had progressed to altered mental status with acute delirium, intermittent tachypnea, and difficulty walking. He recalled a tick crawling on his body during outdoor activities in eastern Oregon within 2 weeks before disease onset. Physical examination revealed temperature of 39.3°C, respiratory rate 22 breaths/min, heart rate 112 bpm, and blood pressure 133/79 mm Hg. The man had difficulty following commands and with word-finding. A transitory macular rash was identified on his trunk on the evening following admission (Figure 1, panel A). 

**Figure 1:**
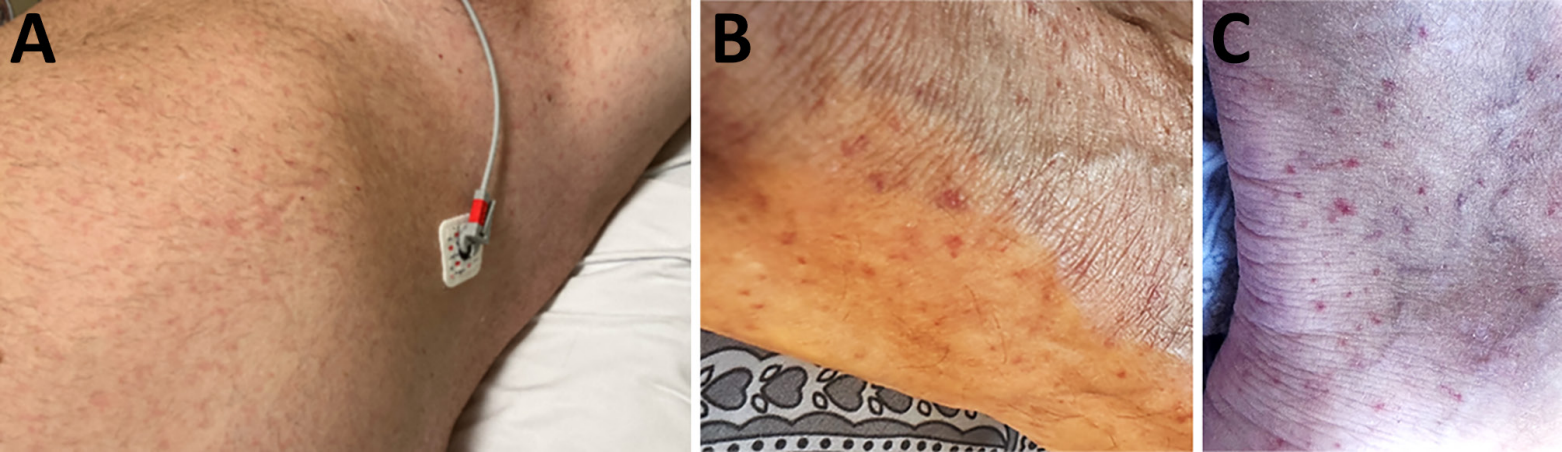
Rash lesions on a patient with *Rickettsia lanei* rickettsiosis, Oregon, USA. A) Pink blanching macules involving the trunk. B, C) Scattered petechiae on the lateral aspect of the foot (B) and posterior aspect of the ankle (C).

Laboratory abnormalities included low serum sodium (124 mEq/L, reference range 135–147 mEq/L), elevated aspartate transaminase (73 U/L, reference range 5–40 U/L), and elevated alanine transaminase (88 U/L, reference range 5–41 U/L). A lumbar puncture was performed; cerebrospinal fluid (CSF) revealed elevated leukocytes (36/µL, reference range 0–5/µL) with 60% lymphocytes and elevated protein (69.3 mg/dL, reference range 15–45 mg/dL). Routine cultures of blood and CSF yielded negative results. PCR and serologic tests for an extensive panel of viral, bacterial, and fungal pathogens also returned negative results, except for IgG (but no IgM) for *Francisella tularensis*.

The patient was treated empirically with intravenous ampicillin, ceftriaxone, and acyclovir. A petechial rash developed on his distal lower extremities (Figure 1, panels B, C). His mental status and transaminase elevations improved over several days, and he became afebrile. He was discharged after 4 days of hospitalization. Plasma was collected at discharge for microbial cell–free DNA (mcfDNA) metagenomic sequencing by using the Karius Spectrum test (Karius, Inc., https://kariusdx.com), which revealed mcfDNA of *R. rickettsii* at 219 molecules/100 nL.

After review by local and state public health officials, and because of the rarity of confirmed RMSF in Oregon, the Oregon Health Authority consulted with the Centers for Disease Control and Prevention, which determined clinical improvement within 11 days in the absence of doxycycline was unusual for classical RMSF. A discussion with medical and scientific staff at Karius, Inc., began and infection with *R. lanei* was considered; however, the recently assembled genome of *R. lanei* (*6*) was not yet publicly available, and the sequenced mcfDNA matched most closely (98%–98.5% average BLAST identity) by research-use only analysis with available genomes of *R. rickettsii* (Figure 2). Subsequent reanalysis using the since-released *R. lanei* genome (GenBank accession no. CP172233) showed 99.2% identity with *R. lanei* (Figure 2). The Centers for Disease Control and Prevention evaluated a convalescent serum specimen obtained 34 days after illness onset and the plasma specimen obtained at discharge by indirect immunofluorescence antibody assay to detect IgG for *R. rickettsii*. Results revealed reciprocal titers of <32 for the discharge specimen and 2,048 from the convalescent specimen.

**Figure 2:**
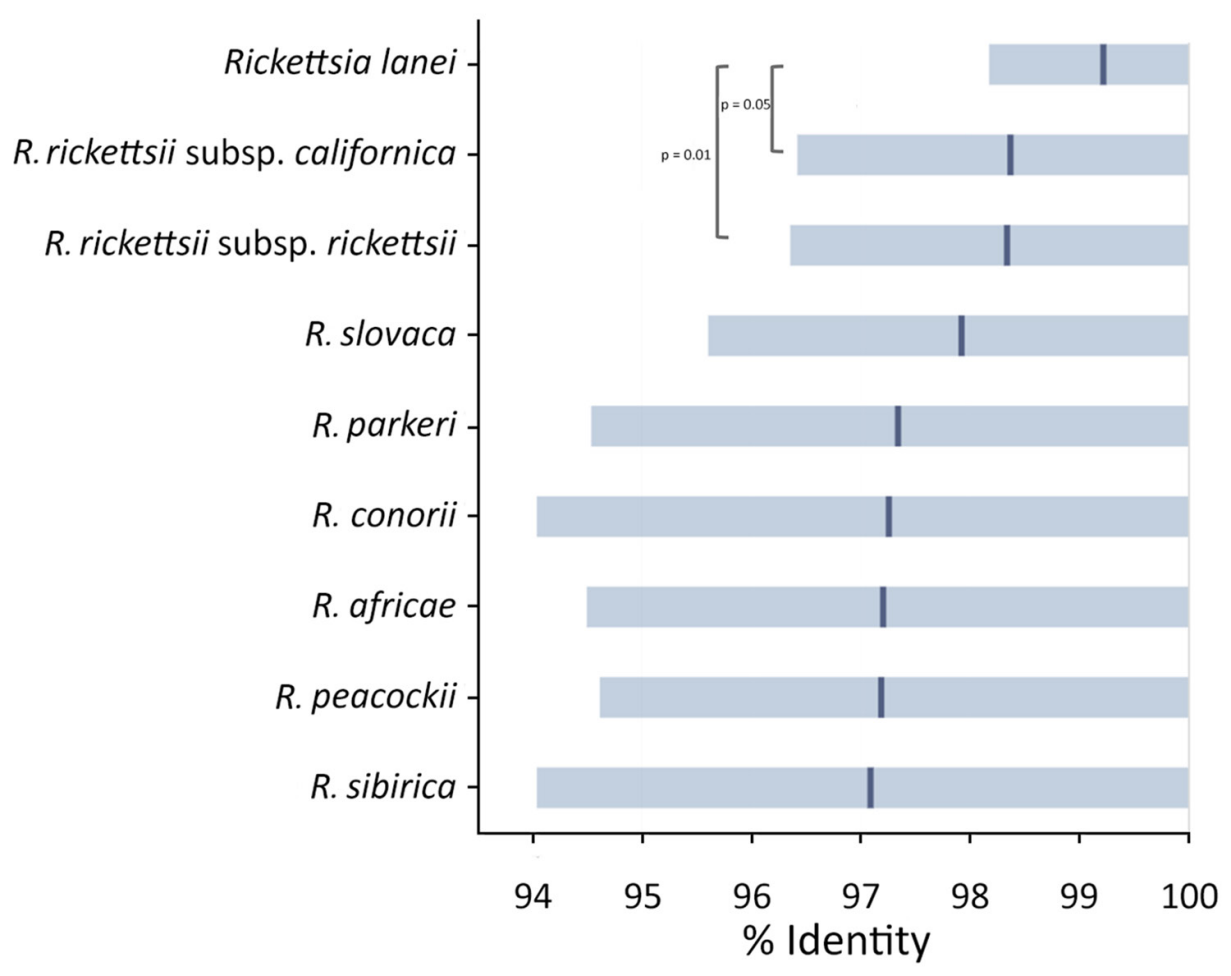
Sequence similarity of microbial cell-free DNA amplified and sequenced from a patient in Oregon, USA, to multiple *Rickettsia* spp. The sequences showed highest sequence similarity (99.2% mean identity) to the recently published *R. lanei* genome assembly (GenBank accession no. CP172233). Means (dark blue lines) and 10th–90th percentiles (light blue shading) of BLAST percentage identity of 967 sequencing reads were calculated for the most similar genome assemblies of related *Rickettsia* species in a custom microbial sequence database. p values indicated for significant differences between *R. lanei* and *R. rickettsii* subsp. *californica* and *R. rickettsii* subsp. *rickettsii*. p values of <10^–6^ were obtained by nonparametric (Wilcoxon signed-rank) and parametric (Student *t*) tests. For each pair, we compared the distribution of percentage identity values, penalized for partial alignment, and with lack of alignment filled in with a subthreshold value of 75%.

Previous case reports of confirmed *R. lanei* rickettsiosis described several clinical characteristics shared with the patient we report, including fever, headache, myalgias, respiratory distress, altered mental status, hyponatremia, and transaminitis (*3*). However, other clinical and laboratory abnormalities identified in previous patients, including nausea, vomiting, respiratory failure, coma, and thrombocytopenia, were not identified in our patient. Rash was documented in our patient but for only 1 of the previously described patients. All 3 patients survived, despite delayed administration of doxycycline, which is uncharacteristic of RMSF (*7*). Those case descriptions suggest that *R. lanei* rickettsiosis can be clinically variable and shares many, but not all, features with classical RMSF.

Wide geographic variations in severity of RMSF have perplexed investigators since first reports of the disease emerged from the western United States >100 years ago. Milder manifestations of RMSF were described from several western states, including Oregon, characterized by lower fever and a substantially lower case-fatality rate compared with classical RMSF (*4*,*5*,*8**–**10*). Other *Rickettsia* species could cause clinically similar diseases of varying severity and thereby account for historically recognized differences in severity described for RMSF in the western United States. Increasingly sensitive molecular detection methods, including mcfDNA sequencing, and access to expanding collections of whole genomes could aid in detection of otherwise unsuspected pathogens and contribute to a more nuanced understanding of SFGR epidemiology. Multidisciplinary partnerships among regional and federal health authorities, clinicians, and diagnostic laboratories led to recognition of *R. lanei* rickettsiosis in Oregon and could leverage identification of cases elsewhere. Clinicians should consider *R. lanei* when diagnosing patients with presumptive RMSF in the western United States, particularly in clinical scenarios that might appear unusual or atypical for classical RMSF.
